# Post-operative depletion of platelet count is associated with anastomotic insufficiency following intrahepatic cholangiojejunostomy: a case–control study from the results of 220 cases of intrahepatic cholangiojejunostomy

**DOI:** 10.1186/1471-2482-14-81

**Published:** 2014-10-16

**Authors:** Takehiro Noji, Takahiro Tsuchikawa, Yuma Ebihara, Toru Nakamura, Kentaro Kato, Joe Matsumoto, Eiichi Tanaka, Toshiaki Shichinohe, Satoshi Hirano

**Affiliations:** 1 Department of Gastroenterological Surgery II, Graduate School of Medicine, Hokkaido University, N15W7, Kita-ku, Sapporo Hokkaido 060-8638, Japan

**Keywords:** Biliary reconstruction, Intrahepatic cholangiojejunostomy, Platelet depletion, Liver regeneration

## Abstract

**Background:**

Post-operative anastomotic insufficiency following major hepato-biliary surgery has significant impacts on the post-operative course. Recent reports have revealed that platelets play an important role in liver regeneration and wound healing. From these experimental and clinical results on platelet function, we hypothesized that post-operative platelet depletion (to <10 × 10^4^/μL) would be associated with delayed liver regeneration as well as anastomotic insufficiency of intrahepatic cholangiojejunostomy. However, little information is available regarding correlations between platelet count and these complications. The purposes of the present study were, firstly, to evaluate the incidence of anastomotic insufficiency following intrahepatic cholangiojejunostomy and, secondly, to evaluate whether platelet depletion represents a risk factor for anastomotic insufficiency in intrahepatic cholangiojejunostomy.

**Methods:**

Participants in this study comprised 220 consecutive patients who underwent intrahepatic cholangiojejunostomy following hepato-biliary resection for biliary malignancies between September 1998 and December 2010. Anastomotic insufficiency was confirmed by cholangiographic demonstration of leakage from the anastomosis using contrast medium introduced via a biliary drainage tube or prophylactic drain placed during surgery.

**Results:**

Anastomotic insufficiency of the intrahepatic cholangiojejunostomy occurred in 13 of 220 patients (6%). Thirteen of the 220 patients, including one with anastomotic insufficiency, died during the study. Uni- and multivariate analyses both revealed that platelet depletion on post-operative day 1 (<10 × 10^4^/μL) correlated with anastomotic insufficiency.

**Conclusion:**

Post-operative platelet depletion was closely associated with anastomotic insufficiency following intrahepatic cholangiojejunostomy. This correlation has been established, but the underlying mechanisms have not.

## Background

Liver resection with intrahepatic cholangiojejunostomy for biliary malignancies is one of the most demanding surgeries and is associated with a high morbidity rate. Recent reports have revealed post-operative morbidity rates ranging from 42% to 59% [1-6]. Morbidity is high because the operations comprise various difficult and highly technical procedures. We have previously reported that the most frequent post-operative complications are organ/space surgical-site infection (SSI), liver failure, respiratory complications, and anastomotic insufficiency of the intrahepatic cholangiojejunostomy [7].

In general, intrahepatic cholangiojejunostomy is thought to be more technically demanding than hepaticojejunostomy. Previous reports have therefore shown that the incidence of anastomotic insufficiency after intrahepatic cholangiojejunostomy (6.8-50%) is higher than that after hepaticojejunostomy (0.4-8%) [4,8-12]. Several studies have reported on bile leakage after intrahepatic cholangiojejunostomy, but those did not clearly specify whether the bile leak arose from the liver stump or from the site of anastomosis for the hepaticojejunostomy, as Nagino et al. suggested in their previous report [9]. Precise determination of the incidence of, and risk factors for, anastomotic insufficiency after intrahepatic cholangiojejunostomy remains difficult.

Recent experimental studies have suggested that blood platelets play a pivotal role in liver regeneration after partial liver resection [13-16]. A depleted platelet count severely suppresses liver regeneration, whereas induction of thrombocytosis by administration of thrombopoietin or splenectomy has been shown to accelerate liver regeneration [14-16]. Some evidence also suggests that platelet-derived serotonin plays an essential role in platelet-mediated liver regeneration [16]. In addition, several clinical studies have revealed that platelet counts below 10 × 10^4^/μL pre-operatively and immediately post-operatively are associated with an increased risk of mortality and delayed recovery of liver function after partial liver resection [17-19]. Experimental evidence has also shown that bile duct cells regenerate along with other hepatocytes [20]. Other recent experimental studies have concluded that platelets play important roles in not only liver regeneration, but also tissue repair [21,22]. Given these results regarding platelet functions, we hypothesized that post-operative platelet depletion (to <10 × 10^4^/μL) would be associated with delayed liver regeneration and anastomotic insufficiency after intrahepatic cholangiojejunostomy. However, data for this hypothesis are lacking. The purpose of this study was therefore to evaluate whether post-operative depletion of platelets was associated with anastomotic insufficiency following intrahepatic cholangiojejunostomy. The primary outcome parameter was incidence of anastomotic insufficiency with intrahepatic cholangiojejunostomy. We performed uni- and multivariate analyses to identify both clinical variables associated with anastomotic insufficiency and clinical factors correlated with platelet depletion in the post-operative period.

## Methods

### Study participants

Participants comprised 220 consecutive patients who underwent intrahepatic cholangiojejunostomy following hepato-biliary resection for biliary malignancies from September 1998 and December 2010.

### Pre-operative biliary drainage

All hepatectomies were performed after the total bilirubin concentration had decreased to below 2 mg/dL. Pre-operative biliary decompression was performed to reduce serum bilirubin concentration to below 2 mg/dL for all patients with jaundice, and to control segmental cholangitis.

### Pre-operative portal vein embolization (PE)

Pre-operative PE of the liver was performed when right trisegmentectomy, right hepatectomy, or left trisegmentectomy was planned. Indications and procedures for PE have been described previously [23].

### Surgery

Hepatectomies were classified according to the Brisbane 2000 Terminology [24]. Hepatoduodenal ligament skeletonization, lymphadenectomy, and various hepatectomies with *en bloc* resection of the caudate lobe and extrahepatic bile duct were performed. Details of these procedures have been described previously [7].

Our procedure for bilioenteric anastomosis using the Roux-en-Y jejunal limb has been described previously with video [25]. Briefly, the jejunal limb was brought to the hepatic ducts via the standard retrocolic-anteduodenal route. In the case of hepatic pancreatoduodenectomy, reconstruction was performed according to Child’s method with an end-to-side pancreaticojejunostomy. All cholangiojejunostomies were performed by anastomosing the mucosal layers of the bile duct and intestine. Transanastomotic stents were placed in all patients, usually introduced via a trans-jejunal route or occasionally a transhepatic route. Although most bile ducts to be anastomosed were drained by percutaneous transhepatic biliary drainage, a trans-jejunal route rather than a transhepatic route was preferred because the sinus tract between the liver and abdominal wall was usually broken at laparotomy.

Drains were placed prophylactically near the anastomosis and the cut surface of the liver. Until the end of 2004, Penrose drains were used along the hepatic stump. From 2005, 10-mm capillary silicone drains were placed in the same spaces.

### Definitions of post-operative complications

Post-operative complications were graded according to the Clavien-Dindo classification [26]. Anastomotic insufficiency of intrahepatic cholangiojejunostomy was identified by cholangiographic demonstration of leakage from the anastomosis using contrast medium introduced via a biliary drainage tube or prophylactic drain placed during surgery [9]. This procedure allowed us to differentiate between an anastomotic leak and bile leaking from the liver stump.

Organ/space SSI was defined as follows: with intra-abdominal collections; drain/s left in place for more than 3 weeks; patients with severe complications such as sepsis requiring admission to the intensive care unit; hemorrhage needing interventional radiology; or repeat laparotomy. In this group, pancreatic- or bile-related infectious complications were also included. Patients with organ/space SSI did not include those with anastomotic insufficiency. Post-operative liver failure was defined as International Study Group of Liver Surgery (ISGLS) liver failure grade B/C [27].

### Statistical analysis

Statistical analyses were performed using the Mann–Whitney U test, a χ^2^ test, or Fisher’s exact test and the Kruskal-Wallis test. Values of *p* <0.05 were considered statistically significant. All variables reaching a *p-*value of less than 0.1 in univariate analysis were included in a multivariate analysis using a logistic step-by-step regression model. Results are presented as odds ratios (ORs) with 95% confidence intervals (CIs).

### Informed consent

The need to obtain informed consent for participation in this retrospective study was waived by the institutional review board.

### Statements

We confirm that all study protocols were approved by ethics committee of Hokkaido University Hospital.

## Results

A total of 220 patients (146 men, 74 women) were enrolled, comprising 140 with extra-hepatic bile duct cancer, 45 with intrahepatic cholangiocarcinoma, and 35 with gallbladder carcinoma. Median age was 68 years (range, 41–81 years).

Pre- and post-operative patient characteristics are presented in Table 1. Median operation time was 647 min (range, 415–1180 min) and median estimated blood loss was 1783 mL (range, 510–24,520 mL). The median number of reconstructed bile ducts was 3 (range, 1–5).

**Table 1:** Subject characteristics

**Subject characteristics**		**Number of subjects**
Age (years) median: 68	>68	126
Sex	Male/Female	146/74
Preoperative diagnosis	BDC	140
	GBC	35
	ICC	45
Body mass index; median: 22	<22	105
Diabetes mellitus	Positive	39
ICGR15 > 10%	Yes	129
ICGR15 > 15%	Yes	37
Preoperative biliary drainage	ENBD/PTBD	64/111
Preoperative albumin (g/dL); median: 3.8	<3.9	112
Preoperative portal vein embolism	Yes	114
Type of hepatectomy	Right	118
	Left	79
	Trisegmentectomy: Right/Left	10/11
	Other	2
Operative blood loss (mL); median: 1783	>1700	108
Operation time (min); median: 647	>660	154
Pancreaticoduodenectomy	Yes	30
Vascular combined resection	Hepatic artery	24
	Portal vein	118
Number of bile duct reconstructions; median: 3	≥3	139
Blood transfusion	Yes	84
Post-operative max AST (IU); median: 443	>440	110
Post-operative max ALT (IU); median: 372	>370	110
Platelet count (pre-operative period)	<12 × 10^4^/μL	6
Platelet count (post-operative day 1)	<10 × 10^4^/μL	30

BDC: extrahepatic cholangiocarcinoma, GBC: gallbladder cancer, ICC: intrahepatic cholangiocarcinoma, ICGR15: indocyanine green retention rate at 15 min, ENBD: endoscopic nasobiliary drainage, PTBD: percutaneous transhepatic biliary drainage, Right: right extended hepatectomy, Left: left extended hepatectomy, max: maximum, AST: aspartate aminotransferase, ALT: alanine aminotransferase.

Anastomotic insufficiency of the intrahepatic cholangiojejunostomy occurred in 13 (6%) of the 220 patients. Other post-operative complications are shown in Table 2. All instances of anastomotic leakage were treated conservatively, by maintenance of a drain that had been placed prophylactically near the cholangiojejunostomy during surgery. One patient died due to post-operative liver failure following anastomotic insufficiency.

**Table 2:** Postoperative complications

**Postoperative complication**	**Number of subjects**
Clavien-Dindo classification IIIa	74
IIIb	5
IVa	8
IVb	0
V	13
Liver failure	28
Anastomotic insufficiency of intrahepatic cholangiojejunostomy	13
Organ/space surgical-site infection	72
Vascular thrombosis	7
Liver abscess	7
Respiratory complications	2
Postoperative bleeding	15

Next, we investigated whether platelet depletion (to <10 × 10^4^/μL on post-operative day (POD)1) was associated with anastomotic insufficiency. According to univariate analysis, platelet depletion was associated with anastomotic insufficiency (p < 0.01), but post-operative liver failure was not. Multivariate analyses revealed that platelet count <10 × 10^4^/μL on POD1 was a significant predictor of anastomotic insufficiency. Vascular combined resection (portal vein and/or hepatic artery) was not associated with anastomotic insufficiency (Table 3).

**Table 3:** Possible risk factors for anastomotic insufficiency following intrahepatic cholangiojejunostomy

			**Univariate analysis**	**Multivariate analysis**		
**Subject characteristics**		**Anastomotic insufficiency (n =13)**	***P***	**Odds ratio**	**95% CI**	***P***
Age (years)	68 > (n =102)	7	*1*			
Sex	Male (n =146)	9	*1*			
	Female (n =74)	4				
BMI	<22 (n =109)	3	*0.083*			*NS*
	>25 (n =33)	1	*0.697*			
Diabetes mellitus	Positive (n =39)	2	*1*			
ICGR15 (%); median: 10	>10 (n =129)	8	*1*			
	>15 (n =38)	3	*0.475*			
Preoperative biliary drainage	ENBD (n =110)	4	*0.262*			
	PTBD (n =64)	6				
	None (n =46)	3				
Preoperative portal vein embolism	Yes (n = 114)	5	*0.396*			
Preoperative PT-INR; median: 1.1	>1.1 (n =75)	4	*1*			
Preoperative albumin (mg/dL)	<3.9 (n = 112)	7	*1*			
Type of hepatectomy	Left lobectomy (n =79)	6	*0.389*			
	Right lobectomy/trisegmentectomy (n =139)	7				
	Other (n =2)	0				
PD	Yes (n =30)	0	*0.224*			
Operative blood loss (mL)	>1700 (n =108)	7	*0.781*			
	>3000 (n =34)	3	*0.429*			
	>5000 (n =13)	2	*0.174*			
Operation time (min)	>660 (n =103)	6	*1*			
Vascular combined resection	Hepatic artery (n =24)	2	*0.639*			
	PV (n =118)	7	*1*			
Number of bile duct reconstructions	≥3 (n =139)	9	*0.772*			
Postoperative max AST (IU)	>440 (n =110)	9	*0.251*			
Postoperative max ALT (IU)	>370 (n =110)	8	*0.569*			
Platelet count (POD1)	<10 x 10^4^/μL (n =30)	5	*0.020*	3.63	1.15-13.01	*0.029*
PT-INR (POD1); median: 1.8	>1.8 (n =105)	5	*0.769*			
APTT (POD1); median: 40	>40 (n =107)	6	*1*			
Postoperative liver failure	Yes (n =28)	3	*0.198*			

BMI: body mass index, DM: diabetes mellitus, ENBD: endoscopic nasobiliary drainage, PTBD: percutaneous transhepatic biliary drainage, PT-INR: prothrombin time-international normalized ratio, PV: portal vein, ICGR: indocyanine green retention rate at 15 min, PD: pancreaticoduodenectomy, AST: aspartate amino transferase, ALT: alanine aminotransferase, POD: postoperative day, N.S: no statistically significant difference.

Finally, we examined clinical factors correlated with platelet count <10 × 10^4^/μL on POD1. According to univariate analysis, platelet counts <10 × 10^4^/μL on POD1 were closely associated with pre-operative platelet count <12 × 10^4^/μL (below the institutional lower limit of normal), operative blood loss >1700 mL, activated partial thromboplastin time (APTT) on POD1 > 40 s, and post-operative organ/space SSI. Among these clinical factors, pre-operative platelet count, operative blood loss, and APTT on POD1 were significant independent predictors of platelet count <10 × 10^4^/μL on POD1 in multivariate analysis (Table 4).

**Table 4:** Correlation between platelet depletion on POD1 and clinical characteristics

**Subject characteristics**			**Univariate analysis**	**Multivariate analysis**		
		**Platelet count**	***P***	**Odds ratio**	**95% CI**	***P***
		**(POD 1)**				
		**<10 × 10**^**4**^**/μL**				
		**N =30**				
Age (years)	>68	19	*0.553*			
BMI	<22	10	*0.116*			
Diabetes mellitus	Yes	8	*0.197*			
ICGR-15	>10%	21	*0.231*			
	>15%	7	*0.430*			
Pre-operative albumin (mg/dL)	<3.9	16	*0.845*			
Pre-operative platelet count	<12 × 10^4^/μL	5	*<0.01*	34.7	3.16-380.2	*<0.01*
Preoperative Portal vein embolism	Yes	20	*0.716*			
Type of hepatectomy	Right/trisegmen-tectomy	21	*0.542*			
PD	Yes	3	*0.775*			
Operative blood loss (mL)	>1700	24	*<0.01*	5.24	1.69-16.2	*<0.01*
Operation time (min)	>660	21	*1*			
Concomitant resection	Hepatic artery	5	*0.339*			
	PV	12	*0.432*			
Number of bile duct reconstructions	>3	14	*0.065*	-	-	*N.S*
Postoperative AST (IU)	>370	15	*1*			
Postoperative ALT (IU)	>440	16	*0.844*			
PT-INR (POD1)	>1.8	18	*0.171*			
APTT (POD1)	>40	26	*<0.01*	5.31	1.71-16.46	*<0.01*
Organ/space SSI (n =30)	Yes	9	*<0.01*	-	-	*N.S*

POD1: post-operative day 1, BMI: body mass index, ICGR-15: indocyanine green retention rate at 15 min), PV: portal vein, Right: right extended hepatectomy, PD: pancreaticoduodenectomy, AST: aspartate amino transferase, ALT: alanine aminotransferase, PT-INR: prothrombin time-international normalized ratio, APTT: activated partial thromboplastin time, SSI: surgical-site infection, N.S: no statistically significant difference.

We verified that patients with pre-operative platelet depletion did not show any significant differences in pre-operative liver functions (prothrombin time-international normalized ratio (PT-INR) or indocyanine green retention rate at 15 min (ICGR-15)). No patients with severe sepsis and platelet depletion underwent hepatectomy.

## Discussion

This study found a 6% incidence of anastomotic insufficiency following intrahepatic cholangiojejunostomy and also revealed that post-operative platelet depletion (to <10 × 10^4^/μL on POD1) might be associated with increased risk of anastomotic insufficiency.

The incidence of anastomotic insufficiency following intrahepatic cholangiojejunostomy is thought to be higher than that with hepaticojejunostomy. Nagino et al. proposed that this was because the bile duct is small and fragile, and multiple anastomoses are often necessary. However, their surgical results for intrahepatic cholangiojejunostomy were suitable, with an anastomotic insufficiency rate of 6.4% (3.6% in the later period with increased experience) [9]. Our operative results with intrahepatic cholangiojejunostomy (anastomotic insufficiency rate, 6%) also appear suitable. Furthermore, Nagino et al. suggested that intra-operative massive hemorrhage or patient age were more closely associated with anastomotic insufficiency than the number of bile ducts reconstructed [9].

The present study identified a different risk factor for anastomotic insufficiency: post-operative platelet depletion. Our data showed operative blood loss >1700 mL was associated with post-operative platelet depletion on univariate analysis (Table 4). Previous results from Nagino et al. that intra-operative massive hemorrhage was associated with anastomotic insufficiency might thus be explained by post-operative platelet depletion [9].

A potentially important determinant of anastomotic insufficiency due to poor wound healing is blood supply, which for the bile ducts are accomplished mainly by the arterial system. We hypothesize that concomitant hepatic artery resection and reconstruction might have been associated with anastomotic insufficiency of intrahepatic cholangiojejunostomy, because these cases had risk factors for thrombosis. Our series included no cases with hepatic artery obstruction in the post-operative period, and our data showed no correlation with hepatic artery combined resection or anastomotic insufficiency (Table 4).

Recent clinical studies have revealed that platelet depletion in the pre- and post-operative periods is associated with delayed liver regeneration [17], and the present data support this.

Alkozai et al. noted that patients with a low immediate post-operative platelet count (<10 × 10^4^/μL) displayed worse post-operative liver function. A low platelet count immediately post-operatively was identified as an independent risk factor for delayed post-operative recovery of liver function [17]. Liver regeneration means not only regeneration of hepatocytes, but also endothelial cells, Kupffer cells, and bile duct cells. Indeed, experimental evidence has suggested that bile duct cells regenerate along with other hepatocytes [20]. Post-operative platelet count may thus be directly related to recovery at the anastomotic site after intrahepatic cholangiojejunostomy.

The mechanism by which platelets participate in liver regeneration may work as follows: contact between platelets and hepatocytes initiates signal transduction involved in growth factor activation. Hepatocyte growth factor (HGF), vascular endothelial growth factor (VEGF), and insulin-like growth factor-1 were also found to contribute to hepatocyte proliferation [2].

Other recent experimental studies have concluded that platelets play an important role in not only liver regeneration, but also tissue repair mechanisms [21,22]. At least 60 different biologically active substances in platelets are involved in tissue-repair mechanisms such as chemotaxis, cell proliferation and differentiation, angiogenesis, intracellular matrix deposition, immune modulation, antimicro-bial activity, and remodeling. Among these, growth factors (epidermal growth factor, platelet-derived growth factor, transforming growth factor alpha, and transforming growth factor beta) are thought to be the most important [22]. Platelet-rich plasma (PRP) has long been used as a source of platelet-related growth factors [21,22]. Several studies using animal models have shown that PRP exerts positive effects on inflammation, remodeling, and increased vascularity after intestinal or bronchial anastomosis [28,29].

The above experimental evidence, along with clinical data (including the present findings), supports the hypothesis that post-operative platelet depletion is closely associated with anastomotic insufficiency following intrahepatic cholangiojejunostomy. Various mechanisms have been thought to be associated with post-operative platelet depletion. Post-operative platelet depletion was commonly attributed to severe systemic infection with disseminated intravascular coagulation or massive hemorrhage. Our data showed that infectious complications were not associated with post-operative platelet depletion (on multivariate analysis), and none of our patients showed sepsis during the early post-operative period (data not shown). Our data do not rule out the possibility that post-operative platelet depletion is associated with intra-operative hemorrhage.

A few limitations to this study must be considered when interpreting our findings. One problem was the limited number of patients with anastomotic insufficiency (n = 13), which rules out identification of strong correlations with any risk factor. A multi-institutional database would allow further discussion of the risk factors for anastomotic insufficiency after hepatic resection for cholangiocarcinoma. However, the present findings represent a first step toward such a multi-institutional study to clarify the risk factors for anastomotic insufficiency following intrahepatic cholangiojejunostomy.

Another problem was that our present study was limited by its retrospective design. We also cannot fully exclude the possibility that patients with a low platelet count on POD1 were simply in a worse general condition, and therefore showed delayed recovery of liver regeneration or a greater incidence of anastomotic insufficiency than patients with normal platelet counts. However, we could not identify any significant differences between patients with and without pre-operative platelet depletion in terms of pre-operative liver function (PT-INR or ICGR-15). The reasons for the association between pre-operative platelet depletion and anastomotic insufficiency following intrahepatic cholangiojejunostomy thus remain unclear. The point clearly identified by our data was that pre-operative platelet depletion and intra-operative massive hemorrhage were both closely associated with post-operative platelet depletion, and post-operative platelet depletion was a significant cause of anastomotic insufficiency following intrahepatic cholangiojejunostomy.

If platelet depletion was a significant cause of anastomotic insufficiency following intrahepatic cholangiojejunostomy, one potential strategy could be to deliberately increase the platelet count during the early post-operative period. However, the current findings should not be seen as a recommendation for more liberal use of platelet concentrates from blood donors, as several studies have shown that platelet transfusion is associated with increased risks of post-operative morbidity and mortality [30-32]. In contrast to endogenous platelets, platelets from blood donors are frequently in an activated state and may induce a range of inflammatory reactions and unwanted side effects [33,34]. New strategies for increasing platelet count safely or for the use of growth factors associated with wound healing and liver regeneration are thus needed. One possible strategy is the use of PRP to decrease anastomotic insufficiency. However, before clinical applications can be seriously contemplated, further investigation is needed to clarify whether PRP is useful for reducing the incidence of anastomotic insufficiency following intrahepatic cholangiojejunostomy. Given our findings and the results from Nagino et al., minimizing intra-operative hemorrhage is important [9].

## Conclusion

The incidence of anastomotic insufficiency following intrahepatic cholangiojejunostomy was 6% in this single, high-volume center experience.

Post-operative platelet depletion (to <10 × 10^4^/μL on POD1) was associated with anastomotic insufficiency following intrahepatic cholangiojejunostomy, but further experimental and clinical studies are needed to elucidate the underlying mechanisms and clarify correlations between platelet count and intrahepatic cholangiojejunostomy.

## Competing interests

The authors declare that they have no competing interests.

## Authors’ contribution

TN and TT participated in the design of the study, performed the statistical analysis, and wrote all parts of the manuscript. YE, TN, KK, JM, and TS participated in coordination and helped to draft the manuscript. ET and SH performed most of the surgery in this series, participated in the design, and also helped to draft the manuscript. All authors read and approved the final manuscript.

## Pre-publication history

The pre-publication history for this paper can be accessed here:

http://www.biomedcentral.com/1471-2482/14/81/prepub
